# Low Serum Asprosin Levels in Fibromyalgia Syndrome: Insights from a Cross-Sectional Study

**DOI:** 10.3390/medicina61030410

**Published:** 2025-02-26

**Authors:** Muhammed Fuad Uslu, Emine Yıldırım Uslu, Sevler Yıldız, Muhammed Fatih Tabara

**Affiliations:** 1 Department of Internal Medicine, Elazığ Fethi Sekin City Hospital, Elazığ 23280, Turkey; 2 Department of Physical Medicine and Rehabilitation, Elazığ Fethi Sekin City Hospital, Elazığ 23280, Turkey; 3 Department of Psychiatry, Elazığ Fethi Sekin City Hospital, Elazığ 23280, Turkey; dr_sevler@hotmail.com; 4 Department of Psychiatry, Fırat University Hospital, Elâzığ 23200, Turkey; fatihtabara@gmail.com

**Keywords:** fibromyalgia, asprosin, anxiety, depression

## Abstract

*Background and Objectives:* This study aimed to evaluate serum asprosin levels in female patients with fibromyalgia syndrome (FM), investigate their associations with clinical parameters such as disease severity, anxiety, and depression, and evaluate the potential of serum asprosin levels as a biomarker for fibromyalgia diagnosis. *Materials and Methods:* A total of 80 participants were included in the study, 40 women aged 18–60 years who were diagnosed with FM according to the American College of Rheumatology (ACR) 2016 criteria and 40 healthy women with similar sociodemographic characteristics to the patient group. All participants were measured for hemograms, biochemistry tests, and serum asprosin levels. Additionally, the Fibromyalgia Impact Questionnaire (FIQ), Beck Anxiety Inventory (BAI), and Beck Depression Inventory (BDI) were administered to the patient group. *Results:* The median asprosin level in the case group was 15.01 (SD = 10.08–31.42), while in the control group it was 31.03 (SD = 25.14–35.7). The asprosin levels in the case group were significantly lower than those in the control group (*p* = 0.001). In contrast, AST, vitamin B12, and folic acid levels were significantly higher in the case group than in the control group. When all participants were evaluated, asprosin levels showed a significant positive correlation with systolic arterial blood pressure (Rho = 0.337, *p* = 0.002) and diastolic arterial blood pressure (Rho = 0.238, *p* = 0.033). A cut-off value of 17.72 ng/mL for asprosin levels in the diagnosis of fibromyalgia demonstrated a sensitivity of 60% and a specificity of 90%. *Conclusions:* Low asprosin levels may serve as a potential biomarker for the diagnosis of fibromyalgia in women.

## 1. Introduction

Fibromyalgia (FM) is a chronic rheumatic condition characterized by widespread body pain, often accompanied by fatigue, stiffness, sleep disturbances, and psychiatric symptoms, all of which significantly impair quality of life [1]. It affects approximately 2–8% of the general population and is more prevalent in women and middle-aged individuals [2]. Despite its high prevalence, the etiology of FM remains unclear. Genetic predisposition, sleep disorders, immune system abnormalities, oxidative stress, neurohormonal imbalances, and psychiatric conditions are thought to contribute to its pathophysiology [3]. Dysfunction of the hypothalamic–pituitary–adrenal (HPA) axis has been linked to the development of FM [4]. Such impairment is also associated with insulin resistance and metabolic syndrome [5] and conditions that exhibit a higher incidence in patients with FM, including obesity, metabolic syndrome, and diabetes mellitus [6,7,8]. Furthermore, insulin resistance can lead to focal cerebral hypoperfusion, a phenomenon observed in FM patients [9]. This connection supports the hypothesis that insulin resistance may play a role in the pathophysiology of FM. Adipokines, molecules involved in the regulation of insulin resistance, have garnered increasing attention for their potential role in the development of FM [10,11,12].

Asprosin, a novel adipokine, is a glucogenic and orexigenic protein synthesized from white adipose tissue during fasting. It is encoded by two exons (exon 65 and 66) of the Fibrillin 1 (FBN1) gene. Asprosin plays a critical role in regulating glucose metabolism, appetite, and insulin resistance through various signaling pathways [13]. During fasting or in obesity, asprosin stimulates glucose production in the liver. According to Romere et al., the glucogenic effect of asprosin occurs independently of glucagon and catecholamine activation. High insulin levels inhibit asprosin-mediated glucose release, while low glucose levels stimulate asprosin production, and high glucose levels suppress it. Asprosin levels are also influenced by the circadian rhythm, with significant increases following overnight fasting and decreases after the first meal of the day [14]. In addition to its glucogenic effect, asprosin has been shown to impair insulin sensitivity in muscle cells, contributing to skeletal muscle insulin resistance [15]. Pathologically elevated asprosin levels have been reported in obesity, insulin resistance, and both type 1 and type 2 diabetes mellitus [16,17,18,19].

According to the literature, overweight, insulin resistance, and obesity are associated with the worsening of FM symptoms [20,21]. In addition to their effects on satiety mechanisms and the preservation of body weight, adipokines are known to have pro- and anti-inflammatory effects and to have pro- and anti-nociceptive properties that regulate pain perception [22]. Recently, the role of adipokines in the development of chronic pain has been focused on [23]. Adipokines such as leptin, asprosin, visfatin, and adiponectin are discussed in relation to pain perception [24]. The role of asprosin in pain modulation in various physiological and pathological processes, especially in neuropathic pain, has recently been discovered [25]. Therefore, it is thought that they may also be related to FM. However, the role of adipokines in the pathophysiology of FM remains largely unknown. Paiva et al. [26] examined leptin and adiponectin levels in FM patients in their study. They observed that leptin levels were lower in the FM group than in controls, while adiponectin levels were similar in both groups. In their study, Ablin et al. [27] observed similar leptin levels in FM patients and controls. We thought that asprosin might be related to FM, that its serum levels might be associated with symptom severity, that it might have diagnostic value in FM, and that it might be preferable to other molecules because of its advantage of being a low-cost, practical measurement. To the best of our knowledge, there is no study in the literature investigating the importance of asprosin in FM. In the present study, we aimed to evaluate the association between serum asprosin levels and disease severity, depression, and anxiety in patients diagnosed with FM.

## 2. Materials and Methods

### 2.1. Participants and Study Design

The patient and control groups were created using a simple sampling method from individuals who applied to the Physical Medicine and Rehabilitation and Internal Medicine outpatient clinics of Fethi Sekin City Hospital between June 2024 and August 2024.

Inclusion criteria for the patient group were determined as patients who met the diagnosis of FM according to the American College of Rheumatology (ACR) 2016 criteria, were between the ages of 18 and 60, were female, had not been previously diagnosed with FM, and had not received any specific treatment in this regard.

#### The ACR Classification Criteria

Fibromyalgia diagnosis was established according to the 2016 American College of Rheumatology (ACR) criteria. The diagnostic criteria included the assessment of the Widespread Pain Index (WPI) and the Symptom Severity Scale (SSS). Patients were considered to have fibromyalgia if they met one of the following conditions: WPI ≥ 7 and SSS ≥ 5 or WPI 4–6 and SSS ≥ 9. The WPI was determined by evaluating pain in 19 predefined body regions, with scores ranging from 0 to 19. The SSS was calculated based on the severity of fatigue, sleep disturbances, and cognitive symptoms, with a total score ranging from 0 to 12. To confirm the diagnosis, symptoms had to persist for at least three months, and alternative medical conditions that could explain the symptoms were excluded. The diagnostic assessment was conducted by experienced clinicians specializing in rheumatology [28].

Exclusion criteria for the patient group were as follows: pregnant women, those with active infection, those with BMI > 30 kg/m^2^, those with a history of inflammatory disease, malignancy, psychiatric or neurological disease, diabetes mellitus, or another endocrinological disease, and those with a history of alcohol or substance use in the last 6 months.

The healthy control group was composed of healthy women who came for regular annual check-ups and did not have any medical problems. Considering the studies conducted with asprosin in the literature and according to the sample calculation we made using the G*power statistical program (GPower_3.1.9.7), the following parameters were employed: effect size: 0.77; power (Power, 1 − β): 0.95; significance level (α): 0.05. As a result of the G*Power analysis, the total minimum sample size required for our study was calculated as 74 (n1 = 37 and n2 = 37) [29], and 47 patients and 43 healthy volunteers who agreed to participate in the study were included in the study. Seven female patients from the FM group and three women from the healthy control group were excluded from the study as they later chose to withdraw.

Participants were initially evaluated by internal medicine and physical medicine and rehabilitation physicians. Subsequently, psychiatrists conducted structured interviews based on the Diagnostic and Statistical Manual of Mental Disorders (DSM-5), each lasting approximately 30 min. After obtaining signed written informed consent, all participants completed the Sociodemographic and Clinical Data Form, the Fibromyalgia Impact Questionnaire (FIQ), the Beck Anxiety Inventory (BAI), and the Beck Depression Inventory (BDI).

### 2.2. Clinical Variables and Blood Biomarker Analysis

Blood samples were collected from all participants between 08:00 and 09:00, after at least 8 h of fasting. Routine biochemistry analysis was performed using an automated analyzer (Beckman AU 5800, Brea, CA, USA). Three milliliters of EDTA-anticoagulated whole blood were utilized for standard hemogram assays (automated hematology analyzer, Beckman Coulter DXH 800, Brea, CA, USA).

The Asprosin (FBN1) ELISA kit (Shanghai Korain Biotech Co., Ltd., Shanghai, China) was employed for analysis. This kit is designed for the detection of human asprosin (FBN1) and operates based on the sandwich enzyme immunoassay principle. The microplate provided with the kit was pre-coated with antibodies specific to FBN1. Serum samples were collected, processed, and stored in accordance with standard laboratory protocols. The kit included all necessary reagents, such as a biotin-conjugated antibody specific to FBN1, Avidin conjugated to Horseradish Peroxidase (HRP), and TMB substrate solution. The assay was performed using a Biotek ELx800 microplate reader (BioTek Instruments, Inc., Winooski, VT, USA). Serum samples were added to the appropriate wells of the microplate, followed by the addition of a biotin-conjugated antibody specific to FBN1. HRP-conjugated Avidin was added to each well, and the plate was incubated to allow for binding. Following incubation, unbound reagents were removed via washing steps. The TMB substrate solution was then added to the wells. Only wells containing FBN1, the biotin-conjugated antibody, and the enzyme-conjugated Avidin exhibited a color change. The enzyme–substrate reaction was terminated by the addition of sulfuric acid solution. The absorbance of the developed color was measured at a wavelength of 450 nm ± 10 nm using the Biotek ELx800 microplate reader.

### 2.3. Scales Used in Study

Sociodemographic and Clinical Data Form: Data on participant age, BMI, marital status, education level, and smoking history were collected.

Beck Anxiety Inventory (BAI): It gauges how frequently the person experiences symptoms of anxiety. An increased level of anxiety is indicated by a higher overall score. Beck et al. [30] created the tool, and Ulusoy et al. [31] proved its validity and reliability in the Turkish population.

Beck Depression Inventory (BDI): It calculates how frequently the person has depression symptoms. A higher overall score indicates a higher level of depression. A validity and reliability study was carried out in our nation, and the instrument was created by Beck et al. [32,33].

Fibromyalgia Impact Questionnaire (FIQ): It was created to evaluate the functional status of patients with FM, and Sarmer et al. assessed its validity and reliability. Higher scores on the FIQ assessment suggest that the disease has a bigger effect on the person [34,35].

### 2.4. Statistical Analysis

Analyses were performed using the SPSS (Statistical Package for the Social Sciences; SPSS Inc., Chicago, IL, USA) version 22 software package. Categorical variables such as marital status, educational status, alcohol use, and smoking were compared using the Pearson Chi-square test. Descriptive statistics were presented using frequencies and percentages for categorical variables and the median and interquartile range (25th–75th percentile values) for continuous variables. The Kolmogorov–Smirnov test, skewness–kurtosis, and histogram graphs were used to evaluate the conformity of continuous variables to normal distribution. The Mann–Whitney U test was used to compare the age, BMI, hemogram, biochemistry, and asprosin values of the groups. The relationship between age, BMI, hemogram, biochemistry parameters, and asprosin was investigated with the Spearman correlation test. The relationship between BDI, BAI, and FIQ scales and asprosin in the case group was also evaluated by using the Spearman correlation test. Bonferroni correction and the False Discovery Rate were applied to eliminate the risk of false positives in multiple comparisons. The ability of asprosin levels to predict the diagnosis of FM was assessed using Receiver Operating Characteristic (ROC) curve analysis. The area under the curve (AUC) was calculated to evaluate the diagnostic accuracy of asprosin levels. The optimal cut-off value for asprosin levels was determined using the Youden index, which balances sensitivity and specificity. Sensitivity, specificity, and AUC values are presented to illustrate the diagnostic performance of asprosin. The ability of asprosin level to predict the diagnosis of FM was investigated by ROC analysis and cut-off values were determined. Whether age, BMI, BDI, BAI, asprosin, insulin, glucose, HbA1c, HOMA-IR, vitamin D, vitamin B12, and folic acid levels predicted FIQ scores was analyzed in a linear regression model. A *p* < 0.05 significance level was accepted for all analyses.

In our study, potential confounding variables such as BMI and vitamin supplementation were addressed through the following methods. Participants with a BMI > 30 kg/m^2^ or conditions affecting metabolic markers (e.g., diabetes, endocrine disorders) were excluded to minimize confounding effects. The patient and control groups were matched for BMI, age, and gender, with no significant differences in BMI observed between the groups. Linear regression and Spearman correlation analyses confirmed that BMI, vitamin B12, and folic acid levels were not significantly associated with fibromyalgia severity or serum asprosin levels. Elevated vitamin B12 and folic acid levels were likely due to prior supplementation. While this was discussed as a potential limitation, no direct relationship between supplementation and asprosin levels was observed. These approaches aimed to minimize confounding effects, though further longitudinal and multicenter studies are recommended for validation. Multivariate regression analysis was performed to exclude the effect of confounding factors on asprosin levels. In this analysis, BMI, HOMA-IR, insulin, vitamin B12, vitamin D, folic acid, and HbA1c were included as potential confounding factors.

### 2.5. Ethical Statement

Ethical approval for this study was obtained from the Firat University Non-Interventional Clinical Research Ethics Committee (date: 27 March 2024; approval number: 23356). The study was conducted in accordance with the ethical principles outlined in the Declaration of Helsinki.

## 3. Results

The study included 80 participants, with 40 women in the case group and 40 women in the control group. The median age of the case group was 40 (IQR = 29.25–45), while the median age of the control group was 29.50 (IQR = 26–45.75) (*p* > 0.05). No significant difference was observed between the groups in terms of BMI (*p* > 0.05). Among the case group, 35 (87.5%) were single, compared to 26 (65%) in the control group (*p* = 0.018). Smoking rates were similar between the groups, with the majority reporting that they did not smoke (*p* > 0.05). No significant difference was found between the groups regarding educational status (*p* > 0.05). A comparison of the sociodemographic data for both groups is presented in Table 1.

The median asprosin level in the case group was 15.01 (IQR = 10.08–31.42), while the median level in the control group was 31.03 (IQR = 25.14–35.7). Asprosin levels in the case group were significantly lower than those in the control group (*p* = 0.001).

Arterial blood pressure, hemograms, and biochemistry values were compared between both groups. Both systolic and diastolic blood pressures were significantly lower in the case group compared to the control group. WBC, hemoglobin, platelet, neutrophil, lymphocyte, monocyte, CRP, and sedimentation values were similar between the groups. However, AST, vitamin B12, and folic acid levels were significantly higher in the case group. Glucose, insulin, HOMA-IR, HbA1c, ALT, urea, creatinine, vitamin D, TSH, and free T4 levels were similar in both groups. A comparison of the blood parameters between the groups is presented in Table 2.

When all participants were evaluated together, a significant positive correlation was found between asprosin levels and both systolic and diastolic arterial blood pressure (Rho = 0.337, *p* = 0.002; Rho = 0.238, *p* = 0.033, respectively). However, no significant correlation was observed between asprosin and other parameters. Additionally, the relationship between asprosin levels and scale scores was investigated only in the case group. No significant correlation was found between the scale scores and asprosin levels. The correlation data for the case group are presented in Table 3.

In the case group, a linear regression model was used to analyze whether age, BMI, BDI, BAI, asprosin, insulin, glucose, HbA1c, HOMA-IR, vitamin D, vitamin B12, and folic acid levels could predict FIQ scores. The analysis revealed that none of these factors had a significant effect (*p* > 0.05).

**Figure 1:**
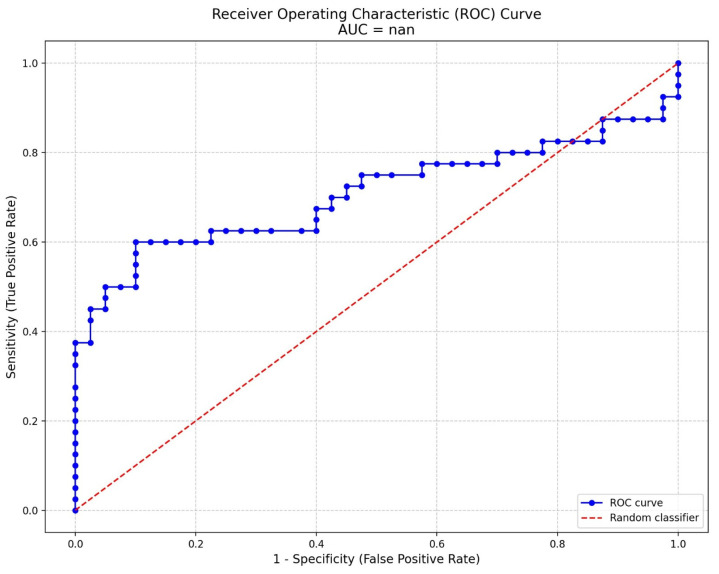
The area under the curve (AUC) is 0.708 (95% CI, 0.586–0.829; *p* = 0.001), indicating moderate diagnostic accuracy of serum asprosin levels for fibromyalgia syndrome (FM). The ROC curve evaluates the trade-off between sensitivity and specificity at various threshold levels. The optimal cut-off value of 17.72 for asprosin levels demonstrates a sensitivity of 60% (95% CI, 51–69) and specificity of 90% (95% CI, 84–96). Clinically, this suggests that while the test may have limited sensitivity, it exhibits high specificity, making it a valuable tool for ruling in FM when asprosin levels are below the cut-off. Further studies are warranted to validate these findings.

Whether asprosin level can be a biomarker for the diagnosis of FM was analyzed by ROC curve analysis (Figure 1). In the ROC analysis, the highest Youden index value (0.5) for asprosin was found to be 17.72 ng/mL. The cut-off value of 17.72 for asprosin level was found to have a 60% sensitivity and a 90% specificity.

BMI, HOMA-IR, insulin, vitamin B12, vitamin D, folic acid, and HbA1c were considered as potential confounding factors. Multivariate regression analysis was performed to observe the effect of confounding factors. The results indicate that the overall model has a low explanatory power (*R*^2^ = 0.056), and none of the independent variables are significant predictors of asprosin levels (*p* > 0.05 for all). These findings suggest that asprosin levels in our study population were not significantly influenced by the included confounding factors.

## 4. Discussion

We investigated the potential of asprosin as a diagnostic biomarker and its relationship with disease severity and psychological distress in patients with FM, a condition for which the etiology remains unclear and for which there are no definitive curative treatments. Our study found that serum asprosin levels were lower in women diagnosed with FM compared to healthy women. However, we did not find a significant correlation between asprosin levels and FM symptom severity, depression, or anxiety levels. We also found that FM patients were more likely to be single, had lower blood pressure than healthy controls, and had significantly higher levels of vitamin B12 and folic acid. A significant positive correlation was found between asprosin levels and both systolic and diastolic arterial blood pressure. Furthermore, our results suggest that serum asprosin levels may serve as a potential diagnostic marker for FM.

A dysfunctional autonomic nervous system has been implicated in FM, with blood pressure reported as an important factor in both functional and cognitive health, particularly in ageing adults. In FM patients, inflammatory mediators are known to affect the peripheral blood circulation, potentially leading to deficiencies in the delivery of nutrients and oxygen to deep tissues. This may promote the accumulation of metabolites and lactic acid, which may explain the deep pain and fatigue symptoms commonly experienced by those diagnosed with FM [36,37,38,39]. In a study of 42 patients and 42 controls, resting blood pressure (both systolic and diastolic) was significantly higher in FM patients compared with controls [37]. Paso et al. compared FM patients with and without depression and found that diastolic blood pressure was lower in FM patients regardless of depression. This was attributed to the fact that blood pressure changes in FM patients were not related to adrenergic effects on contractility and vascular tone, but rather to chronotropic cardiac control [40]. We observed that women diagnosed with FM were more hypotensive than those in the healthy control group. This finding is consistent with some publications in the literature. However, further studies are needed to determine whether hypotension is a consequence or a cause of FM, as previously suggested. In addition, a positive correlation between serum asprosin levels and systolic and diastolic blood pressure was observed in our study. Asprosin is known to increase blood pressure by acting on the paraventricular nucleus through sympathetic activation, and the results of our study are consistent with the literature [41]. In addition, the potential role of vitamins in the severity of symptoms and overall pain in women with FM is well documented [42]. We found that vitamin B12 and folic acid levels were significantly higher in FM patients compared with controls. In contrast, another study found no significant difference in B12 and folic acid levels between FM patients and controls [43]. It is possible that these patients used vitamin supplements to relieve their symptoms before being diagnosed with FM. However, it appears that vitamin supplements alone are not enough to relieve their symptoms. As a result, patients continue to seek further treatment. This highlights the need to clarify the pathogenesis of FM and to develop treatment strategies based on these underlying mechanisms.

Biomarker studies are being conducted to enable early diagnosis of FM, a disease that requires extensive testing for diagnosis and has a significant impact on quality of life. These studies also aim to explore the relationship between biomarkers and disease severity. Components related to inflammation, microinflammation, gut microbiota, oxidative stress, and metabolic syndrome have been the focus of FM biomarker research [44]. Recently, metabolic markers have received increasing attention. One such marker, irisin, a myokine, is a hormone thought to play an active role in the prevention and treatment of obesity and metabolic syndrome. Exercise is the most important factor in stimulating irisin, with the type, intensity, and duration of exercise potentially influencing irisin levels [45,46]. However, studies have found no significant difference in irisin levels between FM patients and healthy controls [47]. Another molecule, nesfatin-1, which is associated with insulin, glucose, and lipid metabolism, was found to be at lower levels in FM patients compared to controls [48]. Asprosin, produced by adipocytes, facilitates glucose production and release [49]. Asprosin is encoded by the fibrillin-1 gene, which also encodes the fibrillin-1 protein. Fibrillin-1 deficiency has been shown to impair wound healing in connective tissue affected by inflammatory diseases [50]. In addition, signals from adipocytes play a crucial role in the function of macrophages, which are involved in inflammation [51]. These findings suggest that asprosin has a promising role as a therapeutic target in metabolic disorders [52]. Given that insulin resistance may be involved in the pathophysiology of FM and that adipokines are molecules associated with the development of insulin resistance [10,11], we examined the relationship between serum asprosin levels and disease severity in patients with FM. We found that serum asprosin levels were lower in women diagnosed with FM than in healthy women, but there was no significant association between FM symptom severity and psychological symptoms. One study suggested that asprosin may regulate the function of mesenchymal stromal cells in improving cardiac function, particularly in myocardial infarction, and explained that asprosin protects against the formation of reactive oxidative metabolites and apoptosis in the ischemic microenvironment [53]. Another study reported that low levels of asprosin had cardioprotective effects under hypoxic conditions by improving mitochondrial respiratory function [54]. Similarly, oxidative stress is known to play a role in the etiopathogenesis of FM, causing permanent damage to cellular enzyme systems through oxygenation disorders [55]. The association between high serum asprosin levels and insulin resistance has been demonstrated in many studies [56]. In our study, while both patients and controls had similar levels of insulin resistance, asprosin levels were lower in FM patients. These findings suggest that FM itself may cause reduced serum asprosin levels independently of metabolic processes and that asprosin levels may be predictive in the diagnosis of FM. However, the finding that symptom severity was not associated with asprosin levels may be due to the fact that symptom severity in FM is associated with many different factors, such as physical activity, sleep quality, psychological factors, inflammatory mediators, cytokines, and serotonin levels [57]. Asprosin may act as a protective molecule against FM due to its ability to protect against oxidative stress. Understanding the mechanism by which asprosin interacts with oxidative stress could potentially lead to the development of targeted medical treatments based on asprosin that may provide more effective relief of FM symptoms.

Our study has several limitations that may affect the generalizability and interpretation of the findings. The limitations of our study include that it was conducted at a single center. Different populations may have different asprosin levels due to genetics, diet, and lifestyle. Conducting the study at a single center may introduce biases due to certain demographic, geographic, or healthcare characteristics of the study population. The cross-sectional nature of the study limits our ability to establish causality. Although we found an association between low serum asprosin levels and FM, the temporal relationship and possible causal mechanisms remain unclear. Whether low asprosin levels contribute to the development of FM or are a consequence of the condition can be determined by longitudinal studies. In addition, the relatively small sample size reduces the statistical power and the ability to detect significant associations. The sensitivity of asprosin as a biomarker in our study was found to be 60%, which is low for clinical application. Therefore, diagnostic accuracy should be supported by further research. In our study, only asprosin was investigated as a biomarker and no comparisons were made with other known biomarkers. Finally, the fact that patients had used various possible vitamin supplements before coming to us may have influenced FM symptom severity and depression and anxiety levels through a placebo effect. The study utilized a convenience sampling method. The sampling method of the study is one of the limitations. We recommend that future studies employ random or stratified sampling techniques to enhance the external validity of the results. Also, the fact that the participants’ dietary habits, medication use (antidepressants, anti-inflammatory therapy, etc.) and physical activity levels were not assessed remains a limitation of our study. Acknowledging these limitations emphasizes the preliminary nature of our results and the need for multicenter, longitudinal studies with larger sample sizes to fully understand the potential role of asprosin as a biomarker for FM.

## 5. Conclusions

As a result, it is vital to diagnose and treat FM patients by addressing both their physical complaints such as pain and fatigue and their mental health issues such as anxiety and depression. In the light of the current findings, we can say that low asprosin levels may serve as a potential biomarker for the diagnosis of fibromyalgia in women. Biomarkers that would enable early diagnosis of fibromyalgia patients may reduce frequent doctor visits due to symptoms and prevent unnecessary investigations. Assessing serum asprosin levels may be an inexpensive and feasible method for diagnosis and for monitoring symptom severity in FM patients. Multicenter and longitudinal studies with larger samples and more diverse populations (taking into account the antidepressant and anti-inflammatory medications used by patients) are warranted to investigate the role of serum asprosin levels as a potential marker in fibromyalgia patients. In fact, comparative studies examining serum asprosin levels in fibromyalgia patients, including other markers such as serum cytokines, cortisol, and serotonin, will be decisive for the use of the asprosin molecule as a diagnostic tool. We think that our study may be a guide for further studies to define fibromyalgia disease and symptom severity.

## Figures and Tables

**Table 1:** Comparison of sociodemographic data and scale scores of case and control groups.

		Case Group (n = 40) n (%)	Control Group (n = 40) n (%)	*p* Value
Age (Median-IQR)		40 (29.25–45)	29.50 (26–45.75)	0.092 ^a^
BDI (Median-IQR)		22 (13–29.75)	-	-
BAI (Median-IQR)		31.50 (17–38)	-	-
FIQ (Median-IQR)		56.16 (42.49–61.82)	-	-
Sex	Woman	40 (%100)	40 (%100)	
Marital status	Single Married	33 (%82.5) 7 (%17.5)	25 (%62.5) 15 (%37.5)	0.045 ^b^
Education status	Primary education High School University	13 (%32.5) 14 (%35) 13 (%32.5)	15 (%37.5) 11 (%27.5) 14 (%35)	0.763 ^b^
Cigarette smoking	Yes No	27 (%67.5) 13 (%32.5)	30 (%75) 10 (%25)	0.459 ^b^

^a^ Mann–Whitney U test. ^b^ Pearson Chi-square test. IQR: interquartile range; BDI: Beck Depression Inventory; BAI: Beck Anxiety Inventory; FIQ: Fibromyalgia Impact Questionnaire.

**Table 2:** Comparison of asprosin, complete blood count, and biochemistry values of case and control groups.

	Case Group (n = 40) (Median-IQR)	Control Group (n = 40) (Median-IQR)	*p* Value
Asprosin, ng/mL	15.01 (10.08–31.42)	31.03 (25.14–35.7)	0.001
BMI, kg/m^2^	25 (22.96–26.09)	24.06 (21.21–25.86)	0.145
Systolic BP, mmHg	111 (100–130)	123.5 (114–135)	0.009
Diastolic BP, mmHg	75.5 (70–81.75)	81 (78–85)	0.008
White blood cell, 10^9^/L	6 (5–7.77)	6.5 (5.42–7.65)	0.507
Hemoglobin g/dL	13.25 (12.5–13.97)	13.35 (12.37–13.9)	1.000
Neutrophil, 10^9^/L	3.28 (2.77–4.58)	3.73 (2.94–4.86)	0.376
Lymphocyte, 10^9^/L	1.9 (1.43–2.46)	1.84 (1.6–2.25)	0.784
Monocyte, 10^9^/L	0.52 (0.41–0.59)	0.46 (0.34–0.55)	0.164
Sedimentation, mm/h	7 (4.25–14.75)	7 (4.25–11)	0.579
CRP, mg/L	2.43 (1.14–5.17)	1.91 (0.94–3.19)	0.229
Fasting blood glucose, mg/dL	87 (82.25–94.75)	88.5 (84–93.75)	0.696
Insulin, µIU/mL	7.99 (5.17–13.24)	7.4 (5.18–10.98)	0.665
HOMA-IR	1.66 (1–2.73)	1.62 (1.09–2.31)	0.700
HbA1c, %	5.55 (5.3–5.7)	5.4 (5.1–5.6)	0.063
AST, IU/L	17.5 (16–20.75)	17 (15–18.75)	0.037
ALT, IU/L	14 (11–18)	13 (11–21)	0.743
Urea, mg/dL	23 (19.25–29.5)	22.5 (20–27)	0.900
Creatinine, mg/dL	0.59 (0.53–0.66)	0.63 (0.59–0.68)	0.054
Vitamin D, µg/L	16 (10.25–24.75)	14.1 (12–22.2)	0.658
Vitamin B12, ng/L	204 (152.5–300.25)	165 (132–222)	0.029
Folic acid, µg/L	8.16 (5.95–11.49)	6.83 (5.55–7.52)	0.003

Mann–Whitney U test. BP: blood pressure; CRP: C-reactive protein; HOMA-IR, homeostasis model assessment of insulin resistance; HbA1c: glycosylated hemoglobin; ALT: alanine aminotransferase; AST: aspartate aminotransferase; IQR: interquartile range; BMI: body mass index.

**Table 3:** Correlation between asprosin levels and scale scores of case group.

	Asprosin Levels	
	Spearman Rho	*p* Value
Beck Depression Inventory	−0.002	0.992
Beck Anxiety Inventory	0.000	0.998
Fibromyalgia Impact Questionnaire	−0.153	0.347

Spearman correlation test.

## Data Availability

The original contributions presented in this study are included in the article. Further inquiries can be directed to the corresponding author.

## Abbreviations

The following abbreviations are used in this manuscript:

FM	fibromyalgia
HPA	hypothalamic–pituitary–adrenal
FBN1	fibrillin-1
ACR	American College of Rheumatology
DSM-5	Diagnostic and Statistical Manual of Mental Disorders
BMI	body mass index
FIQ	Fibromyalgia Impact Questionnaire
BAI	Beck Anxiety Inventory
BDI	Beck Depression Inventory
ELISA	Enzyme-Linked Immunosorbent Assay
SPSS	Statistical Package for the Social Sciences
IQR	interquartile range
BP	blood pressure
HOMA-IR	homeostasis model assessment of insulin resistance
HbA1c	glycosylated hemoglobin

## References

[1] Kocyigit B.F., Akyol A. (2022). Fibromyalgia syndrome: Epidemiology, diagnosis and treatment. Reumatologia.

[2] Borchers A.T., Gershwin M.E. (2015). Fibromyalgia: A Critical and Comprehensive Review. Clin. Rev. Allergy Immunol..

[3] Ruschak I., Montesó-Curto P., Rosselló L., Aguilar Martín C., Sánchez-Montesó L., Toussaint L. (2023). Fibromyalgia Syndrome Pain in Men and Women: A Scoping Review. Healthcare.

[4] Okifuji A., Donaldson G.W., Barck L., Fine P.G. (2010). Relationship between fibromyalgia and obesity in pain, function, mood, and sleep. J. Pain.

[5] Ursini F., Naty S., Grembiale R.D. (2011). Fibromyalgia and obesity: The hidden link. Rheumatol. Int..

[6] Okifuji A., Bradshaw D.H., Olson C. (2009). Evaluating obesity in fibromyalgia: Neuroendocrine biomarkers, symptoms, and functions. Clin. Reumatol..

[7] Yanmaz M.N., Mert M., Korkmaz M. (2012). The prevalence of fibromyalgia syndrome in a group of patients with diabetes mellitus. Rheumatol. Int..

[8] Loevinger B.L., Muller D., Alonso C., Coe C.L. (2007). Metabolic syndrome in women with chronic pain. Metabolism.

[9] Frosch O.H., Yau P.L., Osorio R.S., Rusinek H., Storey P., Convit A. (2017). Insulin resistance among obese middle-aged is associated with decreased cerebrovascular reactivity. Neurology.

[10] Lee Y.H., Song G.G. (2024). Association of circulating leptin, growth hormone, and ghrelin with fibromyalgia: A meta-analysis. Growth Horm. IGF Res..

[11] Achenbach J., Rhein M., Glahn A., Frieling H., Karst M. (2022). Leptin promoter methylation in female patients with painful multisomatoform disorder and chronic widespread pain. Clin. Epigenetics.

[12] Gamberi T., Magherini F., Fiaschi T. (2019). Adiponectin in Myopathies. Int. J. Mol. Sci..

[13] Lee T., Yun S., Jeong J.H., Jung T.W. (2019). Asprosin impairs insulin secretion in response to glucose and viability through TLR4/JNK-mediated inflammation. Mol. Cell Endocrinol..

[14] Romere C., Duerrschmid C., Bournat J., Constable P., Jain M., Xia F., Saha P.K., Del Solar M., Zhu B., York B. (2016). Asprosin, a Fasting-Induced Glucogenic Protein Hormone. Cell.

[15] Jung T.W., Kim H.C., Kim H.U., Park T., Park J., Kim U., Kim M.K., Jeong J.H. (2019). Asprosin attenuates insulin signaling pathway through PKCδ-activated ER stress and inflammation in skeletal muscle. J. Cell Physiol..

[16] Ugur K., Aydin S. (2019). Saliva and Blood Asprosin Hormone Concentration Associated with Obesity. Int. J. Endocrinol..

[17] Alan M., Gurlek B., Yilmaz A., Aksit M., Aslanipour B., Gulhan I., Mehmet C., Taner C.E. (2019). Asprosin: A novel peptide hormone related to insulin resistance in women with polycystic ovary syndrome. Gynecol. Endocrinol..

[18] Groener J.B., Valkanou A., Kender Z., Pfeiffenberger J., Kihm L., Fleming T., Nawroth P.P., Kopf S. (2019). Asprosin response in hypoglycemia is not related to hypoglycemia unawareness but rather to insulin resistance in type 1 diabetes. PLoS ONE.

[19] Zhang L., Chen C., Zhou N., Fu Y., Cheng X. (2019). Circulating asprosin concentrations are increased in type 2 diabetes mellitus and independently associated with fasting glucose and triglyceride. Clin. Chim. Acta.

[20] Kim C.H., Luedtke C.A., Vincent A., Thompson J.M., Oh T.H. (2012). Association of body mass index with symptom severity and quality of life in patients with fibromyalgia. Arthritis Care Res..

[21] Timmerman G.M., Calfa N.A., Stuifbergen A.K. (2013). Correlates of body mass index in women with fibromyalgia. Orthop. Nurs..

[22] Fantuzzi G. (2005). Adipose tissue, adipokines, and inflammation. J. Allergy Clin. Immunol..

[23] Ozcan M., Ayar A. (2024). Endocrine Aspects of Pain Pathophysiology: Focus on Adipose Tissue. Neuroendocrinology.

[24] Clemente-Suárez V.J., Redondo-Flórez L., Beltrán-Velasco A.I., Martín-Rodríguez A., Martínez-Guardado I., Navarro-Jiménez E., Laborde-Cárdenas C.C., Tornero-Aguilera J.F. (2023). The Role of Adipokines in Health and Disease. Biomedicines.

[25] Ozcan S., Kelestemur M.M., Hekim M.G., Bulmus O., Bulut F., Bilgin B., Canpolat S., Ozcan M. (2022). Asprosin, a novel therapeutic candidate for painful neuropathy: An experimental study in mice. Naunyn-Schmiedeberg’s Arch. Pharmacol..

[26] Paiva E.S., Andretta A., Batista E.D., Lobo M.M.M.T., Miranda R.C., Nisihara R., Schieferdecker M.E.M., Boguszewski C.L. (2017). Serum levels of leptin and adiponectin and clinical parameters in women with fibromyalgia and overweight/obesity. Arch. Endocrinol. Metab..

[27] Ablin J.N., Aronov N., Shimon I., Kanety H., Pariente C., Aloush V., Elkayam O., Levartovsky D. (2012). Evaluation of leptin levels among fibromyalgia patients before and after three months of treatment, in comparison with healthy controls. Pain Res. Manag..

[28] Wolfe F., Clauw D.J., Fitzcharles M.A., Goldenberg D.L., Häuser W., Katz R.L., Mease P.J., Russell A.S., Russell I.J., Walitt B. (2016). 2016 Revisions to the 2010/2011 fibromyalgia diagnostic criteria. Semin Arthritis Rheum..

[29] Gozel N., Kilinc F. (2021). Investigation of plasma asprosin and saliva levels in newly diagnosed type 2 diabetes mellitus patients treated with metformin. Endokrynol. Polska.

[30] Beck A.T., Epstein N., Brown G., Steer R.A. (1988). An inventory for measuring clinical anxiety: Psychometric properties. J. Consult. Clin. Psychol..

[31] Ulusoy M., Şahin N., Erkmen H. (1998). Turkish version of the Beck Anxiety Inventory: Psychometric properties. J. Cogn. Psychother..

[32] Beck A.T., Ward C.H., Mendelson M., Mock J., Erbaugh J. (1961). An inventory for measuring depression. Arch. Gen. Psychiatry.

[33] Hisli N. (1989). Beck depresyon envanterinin üniversite öğrencileri için geçerliği ve güvenirliği. Türk Psikol. Dergisi..

[34] Burckhardt C.S., Clark S.R., Bennett R.M. (1991). The fibromyalgia impact questionnaire: Development and validation. J. Rheumatol..

[35] Sarmer S., Ergin S., Yavuzer G. (2000). The validity and reliability of the Turkish version of the Fibromyalgia Impact Questionnaire. Rheumatol. Int..

[36] Kircher J.A., Cherry B.J., Zettel-Watson L. (2021). Blood pressure as a predictor of everyday cognitive function in aging adults with and without fibromyalgia. Neuropsychol. Dev. Cogn. B Aging Neuropsychol. Cogn..

[37] Kulshreshtha P., Gupta R., Yadav R.K., Bijlani R.L., Deepak K.K. (2012). A comprehensive study of autonomic dysfunction in the fibromyalgia patients. Clin. Auton. Res..

[38] Katz D.L., Greene L., Ali A., Faridi Z. (2007). The pain of fibromyalgia syndrome is due to muscle hypoperfusion induced by regional vasomotor dysregulation. Med. Hypotheses.

[39] Gyorfi M., Rupp A., Abd-Elsayed A. (2022). Fibromyalgia Pathophysiology. Biomedicines.

[40] Reyes Del Paso G.A., Contreras-Merino A.M., de la Coba P., Duschek S. (2021). The cardiac, vasomotor, and myocardial branches of the baroreflex in fibromyalgia: Associations with pain, affective impairments, sleep problems, and fatigue. Psychophysiology.

[41] Wang X., Wang J.X., Chen J.L., Hao W.Y., Xu W.Z., Xu Z.Q., Jiang Y.T., Luo P.Q., Chen Q., Li Y.H. (2022). Asprosin in the Paraventricular Nucleus Induces Sympathetic Activation and Pressor Responses via cAMP-Dependent ROS Production. Int. J. Mol. Sci..

[42] Correa-Rodríguez M., Rueda-Medina B., Casas-Barragán A., Tapia-Haro R.M., Molina F., Aguilar-Ferrándiz M.E. (2021). Dietary Intake Assessment, Severity of Symptoms, and Pain in Women with Fibromyalgia. Clin. Nurs. Res..

[43] Hıra S., Karataş H.G., Haskul İ., Gündüz R., Üstün B., Akyüz M. (2018). Evaluatıon Of Relatıonshıp Between Paın Score And Serum Vıtamın B12 And Folate Levels In Fıbromyalgıa Patıents. Balıkesir Med. J..

[44] Russo M., Santarelli D., Georgius P., Austin P.J. (2024). A Review of Etiological Biomarkers for Fibromyalgia and Their Therapeutic Implications. Pain Physician.

[45] Akbulut T., Cinar V., Aydin S., Yardim M. (2022). The role of different exercises in irisin, heat shock protein 70 and some biochemical parameters. J. Med. Biochem..

[46] Bengin E., Kırtepe A., Çınar V., Akbulut T., Russo L., Aydemir İ., Yücedal P., Aydın S., Migliaccio G.M. (2024). Leptin, Ghrelin, Irisin, Asprosin and Subfatin Changes in Obese Women: Effect of Exercise and Different Nutrition Types. Medicina.

[47] Samanci R., Ataoglu S., Ozsahin M., Ankarali H., Admis O. (2019). An investigation of serum irisin levels and inflammatory markers in fibromyalgia syndrome. N. Clin. Istanb..

[48] Bilgici B., Akyol Y., Ulus Y., Ürkmez S.S., Kuru Ö. (2020). Is there any association between low level of serum nesfatin-1 and fibromyalgia syndrome?. Arch. Rheumatol..

[49] Mazur-Bialy A.I. (2021). Asprosin-A Fasting-Induced, Glucogenic, and Orexigenic Adipokine as a New Promising Player. Will It Be a New Factor in the Treatment of Obesity, Diabetes, or Infertility? A Review of the Literature. Nutrients.

[50] Handa K., Abe S., Suresh V.V., Fujieda Y., Ishikawa M., Orimoto A., Kobayashi Y., Yamada S., Yamaba S., Murakami S. (2018). Fibrillin-1 insufficiency alters periodontal wound healing failure in a mouse model of Marfan syndrome. Arch. Oral. Biol..

[51] Mouton A.J., Li X., Hall M.E., Hall J.E. (2020). Obesity, Hypertension, and Cardiac Dysfunction: Novel Roles of Immunometabolism in Macrophage Activation and Inflammation. Circ. Res..

[52] Moradi N., Fouani F.Z., Vatannejad A., Bakhti Arani A., Shahrzad S., Fadaei R. (2021). Serum levels of Asprosin in patients diagnosed with coronary artery disease (CAD): A case-control study. Lipids Health Dis..

[53] Zhang Z., Tan Y., Zhu L., Zhang B., Feng P., Gao E., Xu C., Wang X., Yi W., Sun Y. (2019). Asprosin improves the survival of mesenchymal stromal cells in myocardial infarction by inhibiting apoptosis via the activated ERK1/2-SOD2 pathway. Life Sci..

[54] Wen M.S., Wang C.Y., Yeh J.K., Chen C.C., Tsai M.L., Ho M.Y., Hung K.-C., Hsieh I.-C. (2020). The role of asprosin in patients with dilated cardiomyopathy. BMC Cardiovasc. Disord..

[55] Castro-Marrero J., Cordero M.D., Sáez-Francas N., Jimenez-Gutierrez C., Aguilar-Montilla F.J., Aliste L., Alegre-Martin J. (2013). Could mitochondrial dysfunction be a differentiating marker between chronic fatigue syndrome and fibromyalgia?. Antioxid. Redox Signal..

[56] Mirr M., Braszak-Cymerman A., Ludziejewska A., Kręgielska-Narożna M., Bogdański P., Bryl W., Owecki M. (2023). Serum Asprosin Correlates with Indirect Insulin Resistance Indices. Biomedicines.

[57] Loçasso F.A., Filho H.A., Alvarenga R.M.P., Schimidt S.L., Fiorelli F.K., Ramos P.d.S., Leidersnaider S.C.L., Blum K., Lewandrowski K.-U., Cunha-Junior E.F. (2024). Assessing the Impact of IL-6 and Serotonin on Pain and Symptomatology in Fibromyalgia: An Exploratory Clinical Study. J. Pers. Med..

